# Neighborhood Landscape Spatial Patterns and Land Surface Temperature: An Empirical Study on Single-Family Residential Areas in Austin, Texas

**DOI:** 10.3390/ijerph13090880

**Published:** 2016-09-02

**Authors:** Jun-Hyun Kim, Donghwan Gu, Wonmin Sohn, Sung-Ho Kil, Hwanyong Kim, Dong-Kun Lee

**Affiliations:** 1 Department of Landscape Architecture and Urban Planning, Texas A&M University, A318A Langford Architecture Center, 3137 TAMU, College Station, TX 77843-3137, USA; dgu@tamu.edu (D.G.); wonmin.sohn@tamu.edu (W.S.); 2 Department of Ecological Landscape Architecture Design, Kangwon National University, Chuncheon 24341, Korea; sunghokil@kangwon.ac.kr; 3 Division of Architecture & Urban Design, Incheon National University, Incheon 406-772, Korea; hwan.kim@inu.ac.kr; 4 Department of Landscape Architecture, Seoul National University, Seoul 151-921, Korea; dklee7@snu.ac.kr

**Keywords:** urban heat island effect, green spaces, land surface temperature, GIS, landscape indices, spatial autocorrelation, FRAGSTATS

## Abstract

Rapid urbanization has accelerated land use and land cover changes, and generated the urban heat island effect (UHI). Previous studies have reported positive effects of neighborhood landscapes on mitigating urban surface temperatures. However, the influence of neighborhood landscape spatial patterns on enhancing cooling effects has not yet been fully investigated. The main objective of this study was to assess the relationships between neighborhood landscape spatial patterns and land surface temperatures (LST) by using multi-regression models considering spatial autocorrelation issues. To measure the influence of neighborhood landscape spatial patterns on LST, this study analyzed neighborhood environments of 15,862 single-family houses in Austin, Texas, USA. Using aerial photos, geographic information systems (GIS), and remote sensing, FRAGSTATS was employed to calculate values of several landscape indices used to measure neighborhood landscape spatial patterns. After controlling for the spatial autocorrelation effect, results showed that larger and better-connected landscape spatial patterns were positively correlated with lower LST values in neighborhoods, while more fragmented and isolated neighborhood landscape patterns were negatively related to the reduction of LST.

## 1. Introduction

Urban green spaces provide neighborhoods with a wide range of health, social, and economic benefits; they improve quality of life, promote physical and mental health, decrease crime rates, and increase property values [1,2,3,4,5,6,7]. However, rapid urbanization has accelerated land use and land cover changes, and modified the structure and function of urban ecosystems [8,9]. The major concern is that urban land conversions with large amounts of impervious structures increase the heat storage capacity of cities and change their microclimates, thus creating the urban heat island effect (UHI). UHI is defined as an isolated urban area where the temperature is relatively higher than the encompassing suburban and rural areas [10]. UHI is of critical importance for substantial climate and health studies to understand the magnitude of its effects [11,12,13,14]. Previous research indicates that the increased heat stress in urban areas has exposed residents to many health problems, including a high concentration level of volatile organic compounds (VOCs) and nitrogen oxides (NO_x_), elevating the incidence of cardiovascular diseases, and heat-related mortality from episodes such as heat stroke [15,16,17,18,19,20,21,22]. In addition, increasing heat in urban areas discourages residents from participating in physical activity due to uncomfortable thermal conditions experienced when walking, bicycling, or undertaking other outdoor activities [23]. Many studies have reported on the importance of built environment conditions that promote physical activity which can prevent health risk factors for chronic diseases, such as cardiovascular disease, diabetes, and obesity [24,25,26].

UHI can be quantitatively assessed by two factors: atmosphere temperature and surface temperature [27]. Atmosphere temperature, as a more direct indicator of UHI, has been used to measure temporal changes through the data obtained from single weather stations or mobile measurements. Conversely, to quantitatively assess surface-based UHI, land surface temperature (LST) has been retrieved from remote-sensed thermal infrared data [28,29,30]. LST distributions are easily mapped for specific spatial units with high coverage, while interpolation is required to map the distribution of air temperature [31,32]. LST captures the energy radiated from diverse structures in built and natural environments in urban areas, including rooftops of buildings, impervious surfaces, and vegetation and water bodies [10,33]. For this reason, it has been used widely to assess the relationships between land cover patterns and the spatial distributions of UHI [8,9,34].

A substantial body of literature has investigated the relationship between green space and UHI, suggesting that urban green spaces have a cooling effect and thus mitigate UHI [8,34,35,36,37,38,39,40]. Studies have reported that the closer and bigger green spaces are, the greater temperature differences exist between green areas and the surrounding built environment [36,40,41,42]. In addition, some literature indicates that green spaces in an urban area with high building height to street width ratios intensified the cooling effect at night as heat trapped inside building canopies was released during night hours [37,38,43,44,45]. Although early studies allowed for the empirical understanding of cooling effects by increased greenness [36,37,41,42,46], they relied on limited observations and simply monitored temperature changes by varying locations inside and outside targeted green areas. Thus, some researchers advanced the methodological approaches to quantify the abundance of greenness and expanded their scope of statistical analyses to seek the spatial relationship between green space and LST. The normalized difference vegetation index (NDVI), a method of measuring the composition of urban green spaces, has been integrated with LST data at a pixel level [47,48,49,50,51]. Since built environments in urban areas limit greenness by including human-built structures, such as roads and buildings, understanding the effect of neighborhood landscape spatial patterns helps to strategically develop mitigation plans for UHI effects at the neighborhood level.

To measure the influences of landscape spatial patterns on distribution of LST, landscape indices have been applied to quantify their density, shapes, and connectivity [9,52,53,54,55,56]. However, more empirical studies are required to explore the role of the spatial patterns of urban landscape in UHI mitigation using neighborhood-level data. The relevant body of literature has focused primarily on simple correlation analysis between landscape indices and LST [8,34,57], and the results have been inconsistent, depending upon spatial and temporal conditions [9,57,58]. To illustrate, Li and his colleagues [8,58] found that the percent cover of green space, mean patch area, and shape index had a negative correlation with LST, while patch density had a positive relationship in bivariate analysis. However, Zhou et al. [9] found that patch density of vegetation showed no significant relationship, while edge density had a negative relationship with LST in multi-linear regression models. Another study suggested that the area-weighted parameter area ratio had a stronger relationship with temperature changes than patch density and shape index [34]. Despite these results, previous researchers emphasize the significance of landscape indices on measuring landscape spatial patterns as well as their impact on increases in urban temperature. Moreover, most of the previous studies examining the relationship between landscape spatial patterns and LST were conducted in major cities in Japan and China [34,55,58,59,60,61,62]; a few empirical studies have investigated the influences of landscape spatial patterns on LST by using cases in the United States [9,63,64,65,66,67]. Though urban dwellers are directly affected by UHI in their living environments, previous studies have typically been conducted on a macroscopic scale (e.g., census track), not a neighborhood scale [8,9,34,57,58]. Utilizing a macroscopic scale has several limits with regard to controlling size and capturing characteristics of particular neighborhoods, and thus comparisons are limited by unit differences when assessing the spatial configurations of neighborhood landscapes.

In sum, a lack of understanding regarding the cooling effects of landscape spatial patterns on neighborhoods limits urban design and planning geared toward strategically mitigating the increasing temperatures faced by neighborhood dwellers. The main objectives of this study, then, were to: (1) assess the relationship between LST and landscape spatial patterns by using multi-regression models after controlling for spatial autocorrelation; and (2) identify the most significant predictor among landscape indices to cooling effects of green spaces at a neighborhood scale.

## 2. Methods

### 2.1. Study Location and Samples

To measure the influence of neighborhood landscape spatial patterns on LST, this study selected 15,862 single-family houses in the city of Austin, Texas, USA. Austin has grown rapidly in recent decades [68] and has relatively varied natural and built environments, including both newly developed and historic neighborhoods, with the Colorado River passing through the city center. Austin has a humid and warm temperate climate; it is located on the borders of sub-tropical humid and sub-humid climate zones. Elevations in the city vary from 120 to 300 m above sea level. Its location gives the city hot summers and relatively mild winters. Based on historical records from 1980 to 2010, the average annual temperature was 20.8 °C, with the highest temperature in August and the lowest in January [69]. The annual temperature typically fluctuates between 5.6 °C and 36.7 °C [70].

**Figure 1:**
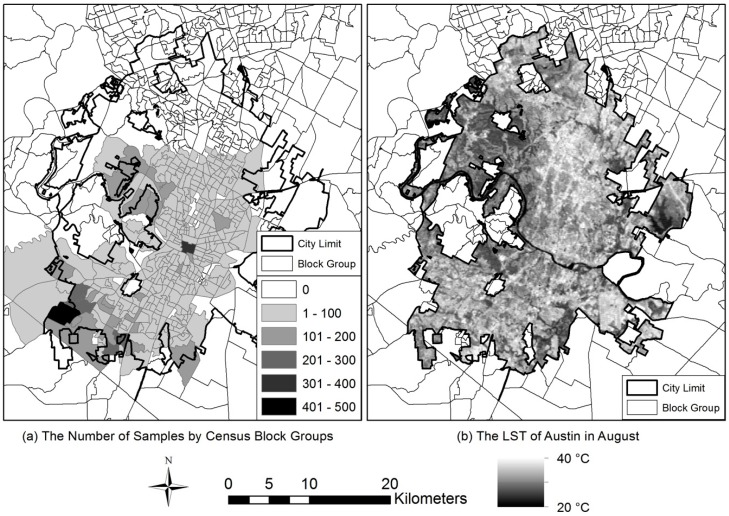
The distribution of the final study samples (**a**) and LST (**b**).

We initially collected 31,670 residential parcel data in Travis County from the Multiple Listing Service (MLS) data provided by the Austin Board of Realtors^®^. From the full dataset, this research eventually selected 15,862 samples of single-family homes after excluding 15,808 samples. Those samples were excluded because they did not show geo-reference points to locate the neighborhood in geographic information systems (GIS) analysis and were listed as multi-family properties. Figure 1 shows: (a) the distribution of the final sample at the census block groups, and (b) the distribution of LST on the research area location in August. The samples were distributed over most urban areas in Austin. The housing density of the study area is 484.06 housing units per one square kilometer, and it is significantly higher than the average density (304.84) of other metropolitan areas in the U.S. The percentage of green space is 14.2% (109.65 km^2^), and it is 0.12 km^2^ per 1000 people. The LST varies within the study area by built and natural environments, and it follows the annual temperature range.

**Figure 2:**
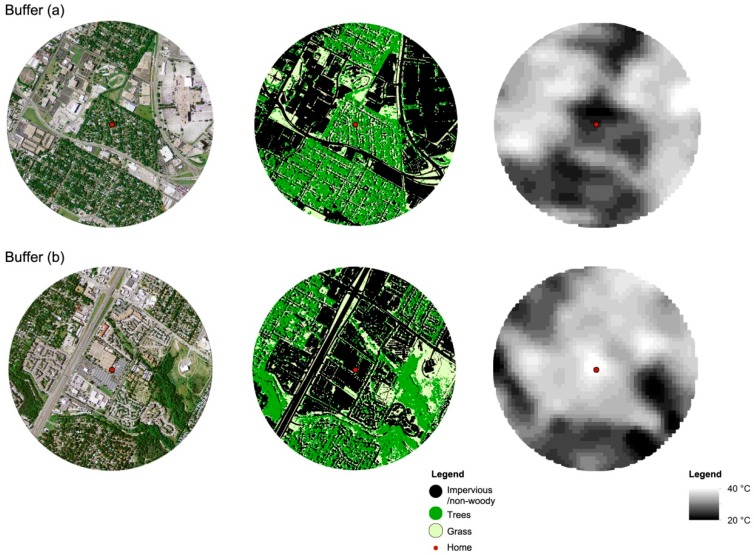
Examples of two buffers measuring LST and neighborhood landscape spatial patterns. (**a**) Example 1; (**b**) Example 2.

To measure landscape spatial patterns in the various neighborhoods based on the final samples, this research used an 800 m Euclidian buffer around each home. The 800 m distance was based on the reported distance of an approximate perceptual and behavioral boundary for a neighborhood. This number has been widely adopted in previous studies measuring neighborhood built environment conditions as a distance that residents are willing to walk in their neighborhoods [7,71,72,73,74] (see Figure 2).

### 2.2. Measuring Landscape Spatial Patterns

Since the quantification of landscape patterns has been highlighted as an area of broad practical interest [75], developing methods to quantify landscape spatial patterns has been emphasized in many previous research efforts [54,75,76,77,78]. This research employed several landscape indices to objectively measure neighborhood landscape spatial patterns. Landscape indices are useful algorithms used to examine specific spatial characteristics of the landscape by acquiring sets of quantitative data [54]. Numerous landscape indices have been developed for monitoring natural resources by evaluating density, complexity, proportion, diversity, proximity, and richness of the specific landscapes. To select appropriate landscape indices measuring the full spectrum of neighborhood landscape spatial patterns, this research applied the five main criteria: size, fragmentation, shape, isolation, and connectivity. These criteria have been developed based on existing principles and guidelines which have been widely applied to ecological planning research [77,79,80,81,82]. Then, six of the most appropriate landscape indices for representing each criterion were selected: percentage of tree cover (PLAND), number of patches (NP), mean patch size (MPS), mean shape index (MSI), mean nearest neighbor distance (MNN), and patch cohesion index (COHESION) (Table 1). PLAND is directly associated with the size of urban forests and tree patches, and higher values of PLAND indicate larger patch sizes. Higher NP and lower MPS values indicate more fragmented patterns of a landscape. MSI measures the shape of patches in urban landscapes. When the MSI value becomes 1, the minimum value, it indicates that the patch has a more regular shape (e.g., a square). MNN examines the distance between the nearest neighboring patches. Higher MNN values mean more isolated landscape patterns. Finally, COHESION values indicate the percentage of physically connected patches. A higher value of COHESION represents a more physically connected landscape spatial patterns.

To analyze the landscape spatial patterns, this study used the Digital Orthophoto Quarter Quadrangles (DOQQ) aerial photos; each had a 1 m high resolution and the photos were taken in 2010. The DOQQ imagery was acquired from the Texas Natural Resource Information System (TNRIS). Using ENVI Version 4.3 (ITT Visual Information Solutions, Boulder, CO, USA), a remote sensing program, we classified the original DOQQ images into 40 land cover types by applying the ISODATA unsupervised classification method. Then, those 40 land cover types were regrouped into three main land cover classes: trees, grasses, and non-woody (impervious) areas. After generating three main land cover classes, this research conducted post-classification processes (sieving, clumping, and filtering) to enhance accuracy of the classifying outcome [83,84]. The classification accuracy assessment was performed using about 52,400 pixels randomly selected from the final classified imagery. The results from the classification accuracy test showed that our classification process was very reliable by reporting high values of accurately classified land cover types. The overall accuracy of the final land cover classification was 95.40% and the Kappa coefficient value was 0.931 (Table 2).

The final classified outcomes were converted into GRID files using ArcGIS Version 10.3 software to capture the neighborhood landscape spatial patterns in 800 m buffers from each house (Figure 2). Finally, FRAGSTATS 4.1 (University of Massachusetts, Amherst, MA, USA), a spatial analysis program developed by McGarigal and Marks [54], was utilized to calculate the value of each landscape index selected at the class level for this research.

### 2.3. Calculating LST

This study assessed the relationship between the neighborhood landscape spatial patterns and the distribution of UHI. Due to a large sample size and distribution of our final sample, we used LST. Mean LST values for each neighborhood buffer were calculated from the calibrated and scaled image data through GIS applications. For these GIS applications, we used ArcGIS and Geospatial Modeling Environment Version 0.7.2.1 [85]. Landsat TM thermal infrared data were acquired from the United States Geological Survey and resampled into 30 m resolution to calculate the LST values [86,87]. The LST image data collected in August 2010 capturing a moment at approximately 12:00 p.m. were selected to prevent time discrepancies between the LST data and the selected DOQQ images. The cloud coverage must be low when data are collected, because clouds reduce variations in LST; thus, this study selected the satellite image data with less than 1% of cloud coverage [88]. For the LST radiometric calibration, Landsat TM/ETM data were given as digital numbers (DNs) ranging between 0 and 255. Equation (1) was used to compute these DN to radiance at the sensor or top-of-atmosphere (TOA) according to radiometric rescaling coefficients [89,90,91]:

(1) $$L_{\left(\lambda \right)}=L_{\min}+\left(L_{\max}-L_{\min}\right)\times \left(Q_{\mathrm{dn}}-\;Q_{min}\right)/Q_{\max}$$

where $L_{\left(\lambda \right)}$ is the spectral radiance at the sensor’s aperture (W·m^−2^·sr^−1^·µm^−1^), $L_{\min}$ is the TOA radiance scaled to $Q_{\min}$ (W·m^−2^·sr^−1^·µm^−1^), $L_{\max}$ is the TOA radiance scaled to $Q_{\max}$ (W·m^−2^·sr^−1^·µm^−1^), $Q_{\mathrm{dn}}$ is the DN value for the analyzed pixel of the TM/ETM image, $Q_{\min}$ is the lowest point of the rescaled radiance in a DN, and $Q_{\max}$ is the highest point of the rescaled radiance in a DN.

To convert spectral radiance to brightness temperature at the sensor, Equation (2) was utilized [89]:

(2) $$T_{i}=\frac{K_{2}}{\ln\left(\frac{K_{1}}{L_{\left(\lambda \right)}}+1\right)}$$

where $T_{i}$ is surface temperature in Kelvins, $L_{\left(\lambda \right)}$ is the computed band radiance from Equation (1) (W·m^−2^·sr^−1^·µm^−1^), $K_{1}$ and $K_{2}$ are calibration constants for Landsat 5 TM, $K_{1}$ = 607.76 (W·m^−2^·sr^−1^·µm^−1^) and $K_{2}$ = 1260.56 K, and for Landsat 7 ETM+, $K_{1}$ = 666.09 (W·m^−2^·sr^−1^·µm^−1^) and $K_{2}$ = 1282.71 K.

### 2.4. Data Analysis

The data analysis for this research focused on examining associations between LST and neighborhood landscape spatial patterns in urban areas. After conducting descriptive statistics, bivariate analyses were performed to mitigate the risk of multicollinearity issues by identifying the correlations between the selected landscape indices. We used the Pearson product-moment correlation coefficients to assess the relationships among variables. After the bivariate analyses, statistical models using a series of the ordinary least square (OLS) regressions were developed to predict the mean LST values as the dependent variable, with the selected landscape indices serving as the independent variables. For the final OLS model (Model 1), the variance inflation factor (VIF) values of the independent variables were utilized to detect potential multicollinearity problems. In addition to the OLS model (Model 1), the spatial lag model (Model 2) was estimated with the maximum likelihood method because the conventional OLS regression method violated the independent observations and uncorrelated error assumptions [92]. Since this research used the mean LST value as a dependent variable, which was spatially correlated with the 800 m neighborhood buffer, the spatial dependency could raise spatial autocorrelation issues. To examine the existence of any spatial autocorrelation effects among the study samples, we conducted the Moran’s I test and found that there existed substantial positive spatial autocorrelations; this meant that similar LSTs were clustered together in the study sample (Moran’s I statistic was 0.73, and *p*-value was less than 0.001). To control for the spatial autocorrelation effects, this study developed a spatial lag model employing the GeoDa Space Version 1.0, a spatial modeling tool that has been used widely in previous studies dealing with spatial autocorrelation issues [93,94].

## 3. Results

Table 3 represents the descriptive statistics for LST, the characteristics of the selected single-family houses, and landscape spatial patterns in each neighborhood. The mean LST, a dependent variable, was approximately 32.60 °C. This indicates the low level of fluctuation in average temperature in August in Austin, Texas. The average housing sale price was about $302,000 ranging from $10,900 to $7,750,000. The living area ranged from 26.66 m^2^ to 1270 m^2^. The homes were approximately 33 years old and had approximately three bedrooms and 2.34 bathrooms, on average.

For the selected landscape indices, approximately 38% of the neighborhood areas within the 800 m buffer were covered by trees (PLAND), and more than 4000 tree and urban forest patches existed (NP), on average. The mean patch size (MPS) was approximately 240 m^2^, and the mean nearest distance (MNN) between two neighboring patches was 2.60 m. Most tree and urban forest patches were highly connected, according to the patch cohesion index (COHESION). Based on bivariate tests, all of the selected landscape indices were significantly correlated with LST. Some landscape indices are highly correlated with each other (the highest value of the correlation coefficient was 0.79 between PLAND and MPS); however, based on the VIF analysis, none of landscape indices showed the risk of multicollinearity. The range of VIF between the selected landscape indices was from 1.51 to 5.45, which was lower than the commonly accepted threshold of 10.

Table 4 shows the final results of this research, displaying both Model 1 (OLS model) and Model 2 (spatial lag regression model). The overall results indicated a strong relationship between LST and the landscape indices. Model 1 explained about 54% of the variance in the relationship between LST and neighborhood landscape spatial patterns (*R*^2^ = 0.5350), while Model 2 explained 82% (*R*^2^ = 0.8201). Based on our final models, the OLS model overstated the relationship between landscape indices and LST. In controlling for spatial autocorrelation issues, while each variable in both the OLS and spatial lag models showed the same signs for dependent variable coefficients, absolute values of the coefficients in the spatial lag model were smaller than absolute values of the coefficients in the OLS model.

The levels of significance for all landscape indices in both of the final models were significant at the 0.01 level. From Model 1, according to the standardized coefficients (Beta) value to compare the explanatory power between independent variables, MPS and MNN were the most significant predictors among landscape indices to cooling effects of trees. The standardized coefficient values of MPS and MNN were −0.396 and 0.353, respectively.

Based on results from Model 2, the percent of tree cover (PLAND) showed a significant negative relationship to LST, which indicates that larger amounts of urban trees and forests in neighborhoods could be positively related to decreased surface temperatures. From measuring fragmentation, it was concluded that the number of patches (NP) was positively associated with LST, while the mean patch size (MPS) showed a significantly negative relationship to LST. These results indicate that more fragmented neighborhood landscape patterns could contribute to an increase in LST. The mean shape index (MSI) was positively related to LST at the 0.01 level, which means that more irregularly shaped neighborhood landscape patterns contributed less to the decrease in LST. The final spatial lag model indicated that there was a statistically negative correlation between the mean nearest neighbor distance (MNN) and LST. This finding suggests that less isolated landscape spatial patterns within an 800 m neighborhood buffer will be likely to reduce LST. Finally, the patch cohesion index (COHESION) measuring the physical connectedness of the tree patches showed a negative relationship; this indicates that well-connected landscape spatial patterns could be more beneficial to decreasing LST.

## 4. Discussion

This study focused on the influences of neighborhood landscape spatial patterns on LST, using landscape indices to quantitatively measure the configurations of landscape structures of neighborhood trees and forests. This study, with a large sample size (approximately 16,000 neighborhood buffers), considered the effects of spatial autocorrelation in order to develop Model 2 (spatial lag regression model) due to the spatial overlap of each unit of analysis. The results from our spatial regression model suggest that larger green spaces (PLAND) and well-connected (COHESION) landscape spatial patterns are positively correlated with lower LST values in neighborhoods, while more fragmented (NP and MPS), irregularly shaped (MSI), and isolated (MNN) conditions are negatively related to the reduction of LST. These results are similar to the findings of previous studies. Li and his colleagues [8,58] found that the percentage of landscape cover and mean patch size were negatively correlated with LST, while patch density showed a positive relationship in their bivariate analysis. Understanding the role of landscape spatial patterns on mitigating LST is important, as the results can be directly translated into urban planning and design guidelines. While previous studies found the proximity, location, and size of green spaces are significantly important factors to reduce temperatures [36,37,41,42,46], those findings are limited to interpolating how the configuration of land cover features can contribute to mitigating LST. Instead of measuring only size and proximity of green spaces, using landscape indices to measure landscape spatial patterns in urban areas allows policy-makers, urban planners, and designers to understand what kind of spatial configurations of neighborhood landscapes should be considered for improving residents’ quality of life and health by reducing LST.

Reducing fragmentation, improving connectivity, and bonding isolated landscape patterns are the most important objectives of ecological planning. Larger patch sizes, well-connected conditions, and less fragmented and isolated landscape patterns are the main indicators of better ecological quality in landscapes [79,80,81]. Following previous theories and guidelines from landscape ecology research, the findings of this study show that ecologically healthy neighborhood landscapes positively contribute to mitigating LST in urban neighborhoods. Our results support that ecological planning should be considered to contribute to environment-public health planning and policy. The current planning framework may miss this connection, which is potentially important to reducing UHI by creating ecologically healthy neighborhood green spaces. The full benefits of neighborhood landscapes shaped by larger and well-connected urban green spaces appear to be significant. Yet, increasing size and enhancing connectivity of green space can often be limited in urban areas due to the existing land use. For compensating the deficiency of urban greenery to mitigate LST, small green spaces as ecological stepping stones can play an important role to increase the size of green space and build green networks in urban areas. Previous studies have also supported the potential ecological contributions of small green spaces to urban planning by assessing school green areas [95], roof gardens [96], and roadside green space [97]. 

Our findings suggest that planning policy and guideline development should consider landscape spatial patterns to improve public health in neighborhoods. Uncomfortable walking and cycling conditions with higher LST tend to keep residents from being involved in outdoor physical activity, which can increase the risk of obesity, cardiovascular diseases, and heat-related mortality [16,17,18,20,21,22,23]. The results of this study imply that if urban forest comprehensive/management plans for neighborhoods would guide larger and well-connected tree areas with less fragmented and isolated patterns, it will likely contribute to reducing LST significantly. This can help to enhance physical and mental health conditions of residents. Improving the quality of life and health of community members is one of the most important goals of urban and landscape planning. As one of the essential elements in neighborhood environments, a better spatial pattern of neighborhood landscapes can enable reduction in LST and should be considered in existing or future planning policies to maximize benefits of allocating and designing neighborhood green spaces.

This study developed two regression models—an OLS model (Model 1) and a spatial lag regression model (Model 2). In Model 2, the effects of spatial autocorrelation were controlled for, which occurred due to the geographical overlap of neighborhood buffers. Both models reported similar estimates for all independent variables. However, Model 2 reduced statistical bias in estimations; the effects of landscape spatial patterns on LST were less exaggerated in Model 2. For example, the influence of the mean nearest neighborhood distance (MNN) and the patch cohesion index (COHESION) on LST was overestimated by about two times in the OLS model. This indicates that if spatial autocorrelation had not been controlled for, it would likely have led to biased outcomes. This result implies that better modeling performances could be achieved when spatial autocorrelation effects are controlled for. Further studies would enhance these findings and suggest solid solutions for healthy neighborhood environments with lower LST.

There are several limitations to this study. First, although we collected a large sample size to analyze the relationships among landscape spatial patterns and LST, the study area was limited to a single city: Austin, Texas. Thus, the findings of this study may not be generalizable to other cities, especially those in geographic areas in the Northern U.S. that have cooler summer temperatures than the study city. In addition, this research used only one land use type, single-family residential areas. Future research should be expanded by estimating the relationship between LST and landscape spatial patterns in diverse land uses including multi-family residential areas. Second, this study employed DOQQ aerial photographs to measure neighborhood landscape spatial patterns. The DOQQ imagery could not detect the full layers of landscape structures with three-dimensional information; it captured only two-dimensional features. Thus, alternative media capable of fully detecting landscape structures under the tree canopy should be considered in future research. Third, this study tested only one size of buffer to measure neighborhood landscape spatial patterns. Future research should perform sensitivity analyses considering different spatial resolution of the data and various scenarios for determining the spatial extension which will improve robustness of estimating results from landscape spatial pattern analysis. In addition, this is a cross-sectional study analyzing the LST data from a selected day. Future research should consider seasonal changes in order to develop more accurate models. Finally, although we selected the most appropriate landscape indices for representing the characteristics of neighborhood landscape spatial patterns, the final model may not fully cover potential control variables such as pavement materials, level of perviousness, and emissivity of neighborhood structures. These factors indicating built environment conditions should be considered to predict more robust estimations when measuring the relationship between urban green spaces and LST.

## 5. Conclusions

This study conducted a spatial regression analysis to examine the relationship between landscape spatial patterns and LST in urban neighborhoods. In the final model, larger green spaces and well-connected landscape spatial patterns positively contributed to reduced LST. In addition, less fragmented and isolated neighborhood landscape patterns were positively associated with lower LST values. Our findings support that ecologically healthier neighborhood landscape patterns are positively correlated to reduced LST values. From the findings of this research, there are several significant contributions to the existing body of literature evaluating UHI. Although there have been a few previous studies exploring the relationship between spatial configurations of green space and LST, the influences of spatial patterns of neighborhood landscapes have not yet been fully investigated. In addition, only few studies have considered the potential spatial autocorrelation issue while analyzing regional-level data, and there have been very few empirical studies focusing on the association between landscape spatial patterns of individual homes and LST in the U.S.

This research will contribute to future investigations by offering evidence useful in determining the extent to which landscape spatial patterns in urban neighborhoods increase in value through the reduction of UHI. Moreover, the findings of this research augment previous studies by considering the use of spatial regression analyses to investigate the role of neighborhood landscape spatial patterns on UHI.

## Figures and Tables

**Table 1:** Selected landscape indices, formulas, and descriptions.

Criteria	Variables (Acronym)	Formula ^a^	Units (Range)
Size	Percentage of tree cover (PLAND)	$\overset{a}{\underset{j=1}{\sum }}a_{ij}/A\times 100$	%
Fragmentation	Number of patches (NP)	$n_{i}$	Count
	Mean patch size (MPS)	$\overset{n}{\underset{j=1}{\sum }}a_{ij}/n_{i}$	Square-meter (MPS ≥ 0, without limit)
Shape	Mean shape index (MSI)	$\left[\overset{n}{\underset{j=1}{\sum }}\left(0.25p_{ij}/\sqrt{a_{ij}}\right)\right]/n_{i}$	None (MSI ≥ 1, without limit)
Isolation	Mean nearest neighbor distance (MNN)	$\overset{a}{\underset{j=1}{\sum }}h_{ij}/n_{i}$	Meter
Connectivity	Patch cohesion index (COHESION)	$\left(1-\overset{n}{\underset{j=1}{\sum }}p_{ij}/\overset{n}{\underset{j=1}{\sum }}\left(p_{ij}\sqrt{a_{ij}}\right)\right)\times {\left(1-1/\sqrt{A}\right)}^{-1}\times 100$	%

Notes: *n*_i_ = number of patches in the landscape of patch type I; *a*_ij_ = area (m^2^) of patch ij; *A* = total landscape area (m^2^); *p*_ij_ = perimeter of patch ij; *h*_ij_ = distance (m) from patch ij to nearest neighboring patch of the same type, based on edge-to-edge distance; ^**a**^ See McGarigal and Marks (1995) for more details. This table is adopted and revised form Kim et al., 2016 [6].

**Table 2:** Classification accuracy assessment.

Classified Class	Reference Pixels (%)				
	Tree	Grass	Impervious Areas	Total	User’s Accuracy
Tree	15,693 (91.26%)	3 (0.02%)	1 (0.01%)	15,697 (29.95%)	99.97%
Grass	366 (2.13%)	16,438 (94.80%)	10 (0.06%)	16,814 (32.08%)	97.76%
Impervious areas	1136 (6.61%)	899 (5.18%)	17,867 (99.94%)	19,902 (37.97%)	89.77%
Total	17,195 (100.00%)	17,340 (100.00%)	17,878 (100.00%)	52,413 (100.00%)	-
Producer’s accuracy	91.26%	94.80%	99.94%	-	-

Overall accuracy = 95.40%; Kappa coefficient = 0.931.

**Table 3:** Summary statistics (*n* = 15,862).

Variables	Mean	SD	Min.	Max.
Land Surface Temperature (LST, °C)	32.60	1.90	23.60	41.40
Landscape spatial characteristics (acronym, unit)				
Percent of tree cover (PLAND, %)	37.98	11.29	3.74	77.53
# of tree patches (NP)	4037.80	1504.03	972	9028
Mean patch size (MPS, m^2^)	239.27	168.77	41.00	1331.00
Mean shape index (MSI)	1.24	0.03	1.15	1.35
Mean nearest neighborhood distance (MNN, m)	2.60	0.43	1.96	6.00
Patch cohesion index (COHESION, %)	99.11	1.08	87.51	99.98

Note: SD = standard deviation; min. = minimum; max. = maximum.

**Table 4:** LST estimation results (*n* = 15,862).

Variables	Coefficients	
	Model 1: OLS ^1^	Model 2: Spatial Lag
**Size**		
Percent of tree cover (PLAND)	−0.0037 ***	−0.0027 ***
**Fragmentation**		
Number of patches (NP)	0.0001 ***	0.0001 ***
Mean patch size (MPS)	−0.0021 ***	−0.0014 ***
**Shape**		
Mean shape index (MSI)	5.2600 ***	3.3966 ***
**Isolation**		
Mean nearest neighbor distance (MNN)	0.7334 ***	0.4715 ***
**Connectivity**		
Patch cohesion index (COHESION)	−0.0916 ***	−0.0590 ***
Constant	306.8238 ***	198.0167 ***
W LST ^2^		0.3546 ***
R-squared (Pseudo R-squared for the Spatial Lag Model)	0.5350	0.8201

Dependent variable: Mean LST (land surface temperature, K) of each neighborhood. ^1^ OLS: ordinary least square; ^2^ W LST: The spatial autoregressive coefficient (spatially lagged dependent variable); *** *p* < 0.01.

## Abbreviations

The following abbreviations are used in this manuscript:

COHESION	patch cohesion index
DN	digital number
DOQQ	Digital Orthophoto Quarter Quadrangles
GIS	Geographic Information Systems
LST	land surface temperatures
MNN	mean nearest neighbor distance
MPS	mean patch size
MSI	mean shape index
NDVI	Normalized Difference Vegetation Index
NP	number of patches
OLS	ordinary least square
PLAND	percentage of tree cover
TNRIS	Texas Natural Resource Information System
TOA	top-of-atmosphere
UHI	urban heat island
VIF	Variance Inflation Factor
